# Lenalidomide restores the osteogenic differentiation of bone marrow mesenchymal stem cells from multiple myeloma patients via deactivating Notch signaling pathway

**DOI:** 10.18632/oncotarget.19265

**Published:** 2017-07-15

**Authors:** Juan Guo, Chengming Fei, Youshan Zhao, Sida Zhao, Qingqing Zheng, Jiying Su, Dong Wu, Xiao Li, Chunkang Chang

**Affiliations:** ^1^ Department of Hematology, Shanghai Jiao Tong University Affiliated Sixth People's Hospital, Shanghai 200233, China

**Keywords:** mesenchymal stem cells, multiple myeloma, osteogenic differentiation, Notch signaling, lenalidomide

## Abstract

Multiple myeloma (MM) always presents osteolytic bone lesions, resulting from the abnormal osteoblastic and osteoclastic function in patients. MM patients exhibit the impairment of osteogenic differentiation of BMMSCs (bone marrow mesenchymal stem cells) and osteoblast deficiency. Effects of the drug, lenalidomide on the osteoblastic functions and the involved mechanisms remain unexplored. In the present study, it is observed that the osteogenic differentiation of BMMSCs from MM patients (MM-MSCs) is impaired and activation of Notch signaling pathway in MM-MSCs is abnormal. Notch signaling activation inhibits BMMSCs osteogenesis. Knockdown of Notch1 expression and DAPT application reverse the osteogenic differentiation from MM-MSCs. Furthermore, it is shown that the gene expression of Notch signaling molecules, including receptors, ligands and downstream factors are significantly decreased in MM-MSCs following lenalidomide treatment, compared with non-treated MM-MSCs. Taken together, treatment with lenalidomide restores the osteogenic differentiation of MM-MSCs via deactivating Notch signaling pathway.

## INTRODUCTION

Multiple myeloma (MM), as a hematological malignancy, is characterized by the abnormal proliferation and presence of clonal plasma cells within the bone marrow. Most patients suffered MM have osteolytic bone disease, leading to severe pain, fractures, spinal cord compression, and hypercalcemia [1–3]. The presence of osteolytic lesions is usually attribute to the imbalance between enhancement of osteoclast activity and attenuation of osteoblast function. The bone marrow mesenchymal stem cells (BMMSCs) are involved in promoting the growth, survival, migration and drug resistance of myeloma cells [4]. Previous reports indicate that BMMSCs from MM patients (MM-MSCs) exhibit the impaired osteogenic differentiation [5–7]. Since MM-MSCs were cultured *in vitro* without MM cells, the diminished osteogenic differentiation of MM-MSCs is not induced by the molecules or factors generated by MM cells rather than due to the intrinsic abnormal cellular molecules or genes. Notch signaling has important roles in the embryonic development and differentiation and the pathway is also necessary for postnatal stage [8–10]. Notch signaling suppresses osteoblast differentiation and keep BMMSCs in the undifferentiated state [11–15]. Therefore, we will explore how Notch signaling suppresses osteogenic differentiation of MM-MSCs, resulting in the diminishment of osteogenic capacity.

Several drugs which reduce bone degradation mediated by osteoclast are applied in clinical. However, the drugs to promote bone formation need to be developed. Novel anti-myeloma drugs not only directly target on myeloma cells and produce the effect on microenvironment as well [16–18]. In previous studies, lenalidomide treatment exhibits an effective intervention for the newly diagnosed and relapsed MM. It is also reported lenalidomide inhibits osteoclast, but the effect of lenalidomide on the osteoblastic function was unconsistent [19, 20].

In the present study, we carried out the gene expression analyses associated with osteogenesis of BMMSCs derived from healthy controls (HC-MSCs) and MM patients (MM-MSCs) and identified the differences between MM-MSCs and HC-MSCs. We also investigated effects of lenalidomide (LEN) treatment on MM-MSCs and explored how Notch signaling suppressed osteogenic differentiation of MM-MSCs, resulting in the impairment of osteogenic capacity.

## RESULTS

### The production of CD271^-^ALP^+^OB (osteoblasts) is lower in MM patients and show negative correlation with severity of disease

**Figure 1:**
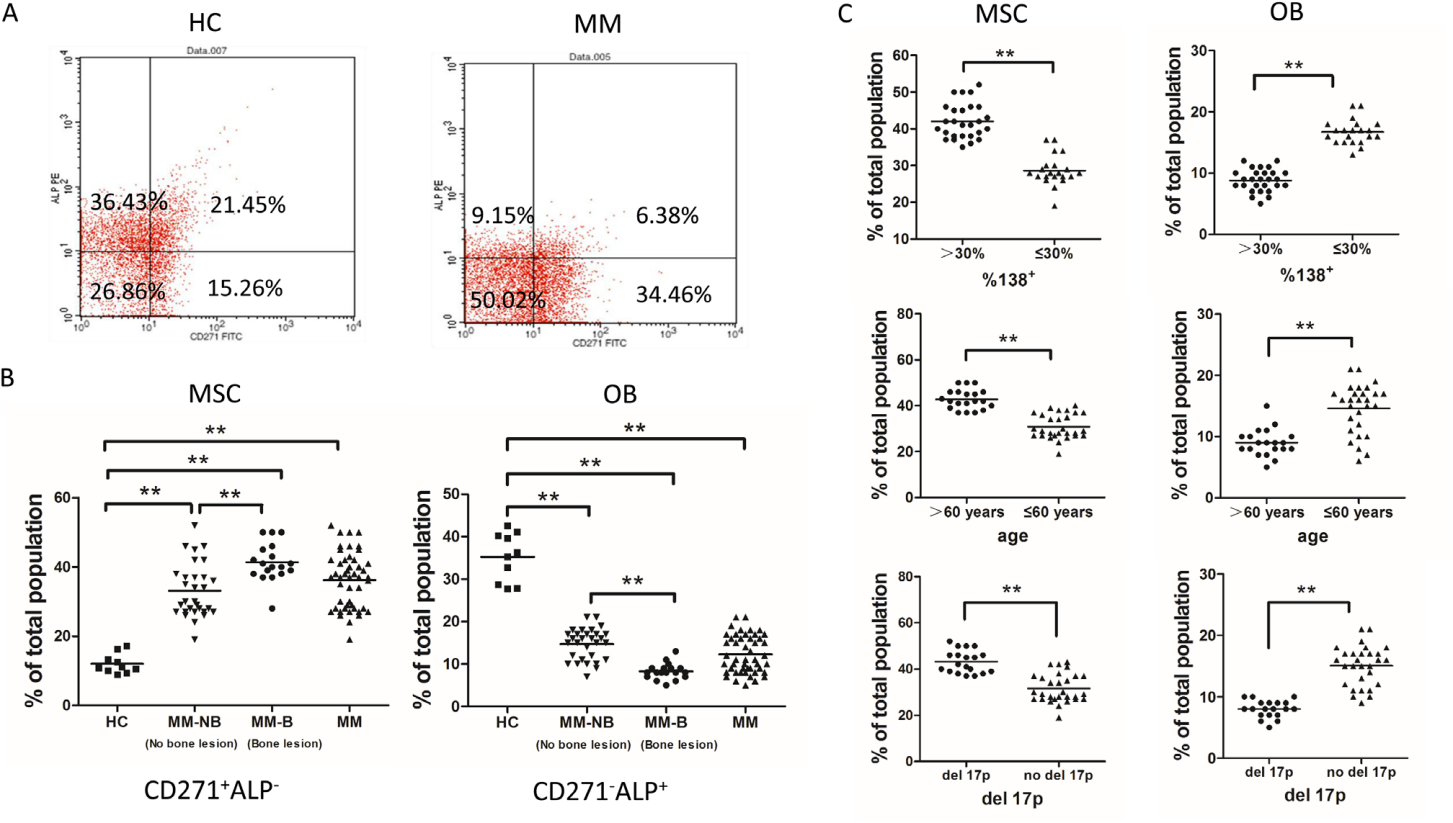
The decreased number of OBs in primary MSCs cultures from MM patients **(A)** CD271-isolated primary MSCs were cultivated for 10 days (3 passages) and were analyzed by flow cytometry for the expression of CD271 and ALP. **(B)** The percentages of CD271^+^ALP^-^ MSC and CD271^-^ALP^+^ OB were compared between healthy controls (HC, n = 10) and MM patients (n=48). The percentage of CD271^-^ALP^+^OB population (of the total population) was significantly lower in patients with osteolytic lesions (MM-B, n=18) than those who exhibited no evidence of bone lesions (MM-NB, n=30). The percentage of CD271^-^ALP^+^OB was significantly lower in MM-NB patients than healthy controls (HC) (**p<0.01). In contrast, the percentage of CD271^+^ALP^-^MSC was significantly higher in MM-B patients than MM-NB patients (**p<0.01). **(C)** CD271^+^ALP^-^MSC (left) and CD271^-^ALP^+^ OB (right) proportion in MM patients based on PC burden in bone marrow (%CD138+), age, and cytogenetics aberrancies. The MM PC infiltration was evaluated by comparing bone marrow from patients with low (≤30% CD138+ cells, n=21) versus high (> 30%, n=27) disease burden. The percentage of CD271^-^ALP^+^OB was significantly lower in patients with high disease burden (>30%, n=27) than those who had lower disease burden(≤30% CD138+ cells, n = 21) (**p<0.01). BMMNC from younger (≤60 years, n=28) subjects contained significantly higher CD271-ALP+ OB and lower CD271+ALP- MSC than that from elder subjects (>60 years, n=20) (**p<0.01). The impact of cytogenetic aberrancies was analyzed by comparing samples from patients with del (17p) (n=19) versus with no del (17p) (n=29). The percentage of CD271^-^ALP^+^OB was lower in MM patients with del (17p) than those who with no del (17p) (**p<0.01). All values are expressed as mean±SD.

Ficoll-density gradient separation was performed to collect mononuclear cells from the bone marrow of MM patients(n=48) and healthy controls (n=10). CD271 is a well-characterized MSC antigen that enriches in the subset of immature, multipotent MSC [21–23]. The CD271^+^ BMMNC were cultured at low density. Explant cultures of mesenchymal stem cells developed from CD271-isolated primary MSCs derived from healthy controls or MM were cultivated for about 10 days (3 passages) *in vitro*. Osteoblasts (OB) and MSC content in the explant cultures were analyzed by dual color flow cytometry through MSC-associated antibodies against CD271, in conjunction with the antibodies against alkaline phosphatase (ALP), a specific marker for OB [21, 24, 25]. As shown in Figure 1A, explant cultures were divided into four populations characterized by the differentiation level of OB, with CD271^+^ALP^-^MSC-like population showing the least differentiation but CD271^-^ALP^+^ OB-like population being the most differentiation. The number of CD271^+^ALP^-^ cells were significantly increased (**p<0.01) in the explant cultures from MM group when compared with that from healthy group. But the number of CD271^-^ALP^+^ osteoblasts was decreased in the explant cultures from MM group (Figure 1B). The percentage of CD271^-^ALP^+^OB population (of the total population) was significantly lower in patients with osteolytic lesions (MM-B, n=18) than those who exhibited no evidence of bone lesions (MM-NB, n=30) (Figure 1B). And the percentage of CD271^-^ALP^+^OB was significantly lower in MM-NB than healthy controls (HC). In contrast, the percentage of CD271^+^ALP^-^ MSC was significantly higher in MM-B patients than MM-NB patients (**p<0.01).

Furthermore, we compared the proportion of CD271^+^ALP^-^ MSC and CD271^-^ALP^+^ OB from MM patients based on plasma cell (PC) burden in bone marrow (%CD138^+^), age, or cytogenetics aberrancies. The MM PC infiltration was evaluated by comparing bone marrow from patients with low (≤ 30% CD138^+^ cells, n = 21) versus high (> 30%, n=27) disease burden. The percentage of CD271^-^ALP^+^OB was significantly lower in patients with high disease burden (>30%, n=27) than those who had lower disease burden(≤30% CD138+ cells, n = 21) (**p<0.01). BMMNC from younger (≤60 years, n=28) subjects contained significantly higher CD271^-^ALP^+^ OB and lower CD271^+^ALP^-^ MSC than that from elder subjects (>60 years, n=20) (**p<0.01). The impact of cytogenetic aberrancies was analyzed by comparing samples from patients with del (17p) (n=19) versus with no del (17p) (n=29). The percentage of CD271^-^ALP^+^ OB was lower in MM patients with del (17p) than those who with no del (17p) (**p<0.01). Collective data indicate the production of CD271^-^ALP^+^OB is lower in MM patients and show negative correlation with severity of disease.

### BMMSCs from MM patients show decreased osteogenic differentiation

**Figure 2:**
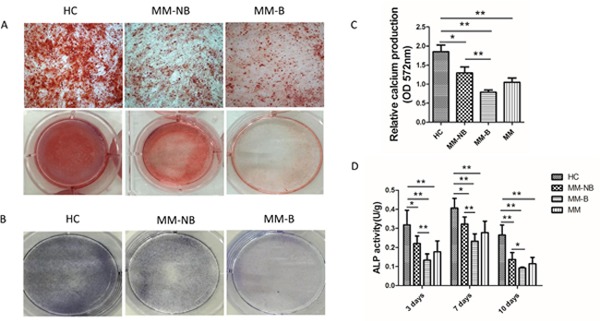
Decreased osteogenic differentiation of MM-MSCs **(A)** After 21 days of differentiation in osteogenic medium (OM), BMMSCs from healthy controls(HC), patients with bone lesion (MM-B) and patients without bone lesion (MM-NB) were identified for the mineralization deposition by alizarin red S staining. Selected micrographs (original magnification, ×100) and original images are shown. **(B)** BMMSCs were evaluated for their osteogenic differentiation potential by ALP staining after 3 days of osteogenic culture. ALP staining was performed with BCIP/NBT. **(C)** After 21 days of differentiation in OM, relative calcium production by BMMSCs from MM patients (MM, n =11) was significantly lower as compared with that form healthy controls (HC, n=10). Additionally, the relative calcium production by MSCs from MM patients with osteolytic lesions (MM-B, n=6) was lower than those from MM patients with no bone lesion (MM-NB, n=5). **(D)** The ALP activity of HC-MSCs (n=6) was significantly higher than that of MM-MSCs (n=20) after 3 days culturing in OM. Furthermore, the ALP activity of MM-B(n=10) was significantly lower than that of MM-NB(n=10). All values are expressed as mean±SD. *p<0.05, **p<0.01.

Next, we investigated the osteogenic differentiation of BMMSCs from MM patients and healthy group. Alizarin red S staining was performed to assess osteoblastic differentiation following 21 days of osteogenic induction. Calcium production by BMMSCs from MM patients was reduced after differentiation for 21 days when compared to healthy controls (Figure 2A, 2C). Additionally, calcium production by MSCs was lower from MM-B than those from MM-NB. And the relative calcium production in the osteogenic medium of MSC was significantly lower from MM-NB than that from healthy controls after 21 days. ALP staining was performed 3 days after osteogenic induction. It was demonstrated that ALP staining was weaker in MSCs from MM patients than that from healthy controls (Figure 2B). ALP activity showed increase with the peak at 7 days after osteogenic induction and then showed reduction (Figure 2D). ALP activity in MM-MSCs was obviously lower than in the cells from healthy group. And it was shown that ALP activity in MSC from MM-B was markedly decreased compared to that from MM-NB.

**Figure 3:**
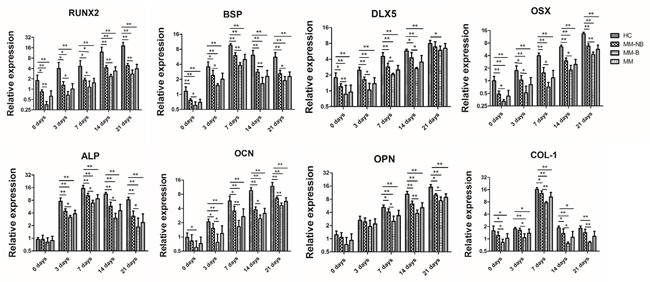
Relative mRNA expression of osteogenic differentiation related gene in MM-MSCs The genes associated with osteogenic differentiation (*RUNX2*, *Osterix*, *BSP*, *ALP*, *COL-1*, *OPN*, *OCN*, *DLX*5) of BMMSCs from HC and MM (MM was distinguished as MM-NB and MM-B) were measured by real-time PCR. At 0, 3, 7, 14, and 21 days of osteogenic culture, the levels of all genes detected were significantly lower in MM (n=18) patients versus healthy controls (HC, n=10). BMMSCs from MM-B (n=8) exhibited lower expression levels of all genes during the whole course of osteogenic induction than those from MM-NB(n=10). All values are expressed as mean±SD. *p<0.05, **p<0.01.

Furthermore, we examined the level of genes involved in osteogenic differentiation in MSCs at 0, 3, 7, 14 and 21 days after the induction of osteogenic differentiation. It was shown an increased gene expression of transcription factor *DLX5*, *RUNX2*, and *OSX* and bone-related factors *OPN* and *OCN*, but the expression of *BSP*, *COL-1*, and *ALP* genes showed the highest level at 7 days and then showed a decrease in MSC from both MM group and healthy group (Figure 3). The expression of all genes investigated exhibited the decrease in MSC from MM patients versus healthy controls, although some markers for OB differentiation (*DLX5* and *COL-1*) did not show statistical significant change between MSC from healthy controls and patients after differentiation for 21 days. Interestingly, MSCs from MM-B exhibited much more lower expression of all genes during the osteogenic induction as compared to those from MM-NB. The above data indicate osteogenic differentiation is decreased in BMMSCs from MM patients.

### Upregulation of the Notch signaling pathway in BMMSCs from MM patients

**Figure 4:**
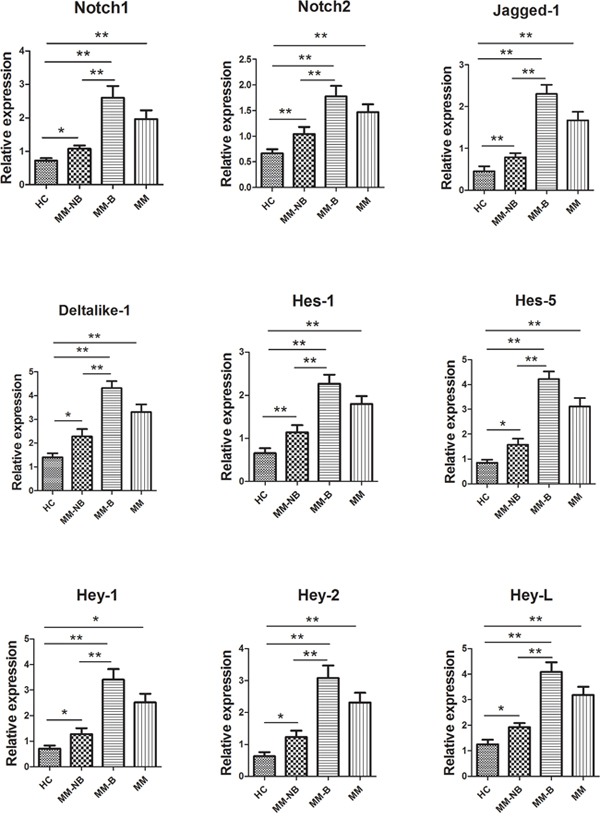
The mRNA expression of genes associated with Notch signaling in BMMSCs from MM patients Real-time PCR was conducted to detect the level of genes involved in Notch signaling pathway. It was shown that mRNA expression of these genes, including Notch receptors *Notch1* and *Notch2*, Notch ligands *Jagged-1* and *Deltalike-1*, and Notch signaling downstream *Hey-1, Hey-2, Hes-1, Hes-5 and Hey-L* was obviously heightened in BMMSCs from MM patients (n=24) compared to that from healthy controls (n=10). The level of these genes was much more higher in BMMSCs from MM-B (n=10) as compared to MM-NB(n=14). All values are expressed as means ± SD. *p < 0.05, **p < 0.01.

To assess whether the impairment of osteogenic differentiation in BMMSCs from MM patients was due to abnormal Notch signaling pathway, we examined the level of genes involved in Notch signaling pathway by RT-PCR, including Notch receptors *Notch1* and *Notch2*, Notch ligands *Jagged-1* and *Deltalike-1*, and Notch signaling downstream *Hey-1*, *Hey-2*, *Hes-1*, *Hes-5* and *Hey-L*. It was shown that the mRNA expression of these genes was obviously heightened in MM-MSCs compared to HC-MSCs (Figure 4). And the level of these genes was much more higher in BMMSCs from MM-B compared with MM-NB (Figure 4).

### Inhibition of Notch signaling by DAPT restores osteogenic differentiation in MM-MSCs

**Figure 5:**
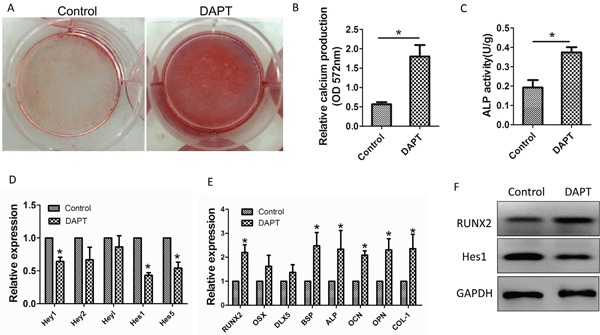
Restored osteogenic differentiation of MM-MSCs by DAPT **(A, B)** Application of 10 μM DAPT in the osteogenic differentiation medium for 48 h increased mineralization evaluated by alizarin red S staining following 21 days of osteogenic induction. **(C)** After 3 days of differentiation in osteogenic medium, ALP activity showed an increase in MM-MSCs treated with DAPT. **(D)** DAPT treatment induced a decreased expression of *Hes-1*, *Hes-5* and *Hey-1* genes in impaired-osteo MM-MSCs. **(E)** The expression of genes, *RUNX2*, *BSP*, *ALP*, *OCN*, *OPN* and *COL-1* involved in osteogenic differentiation showed higher level in impaired-osteo MM-MSCs cultured in osteogenic induction medium in addition with DAPT for 7 days. **(F)** DAPT treatment decreased the expression of Hes-1 but increased the expression of RUNX2 in BMMSCs from impaired-osteo MM patients. Each experiment was repeated three times. All values are expressed as means ± SD. *p < 0.05.

To confirm the necessity of Notch signaling for the osteogenic commitment of MM-MSCs. The γ-secretase inhibitor, DAPT was applied to inhibit the activation of Notch signaling. Application of 10 μM DAPT in the medium for 48 h decreased the expression of *Hes-1*, *Hes-5* and *Hey-2* genes in impaired-osteo MM-MSCs (Figure 5D). And as shown in Figure 5E, the expression of genes, *RUNX2, BSP, ALP, OCN, OPN and COL-1* involved in osteogenic differentiation showed higher level in impaired-osteo MM-MSCs cultured in osteogenic induction medium in addition with DAPT for 7 days. DAPT treatment induced a decreased expression of *Hes-1*, *Hes-5* and *Hey-1* genes in impaired-osteo MM-MSCs. Western blot results showed that DAPT treatment decreased the expression of Hes-1 but increased the expression of RUNX2 in BMMSCs from impaired-osteo MM patients. (Figure 5F). Furthermore, impaired-osteo MM-MSCs showed the increased mineralization following treatment with DAPT after osteogenic induction for 21 days and the enhanced ALP activity after osteogenic induction for 3 days (*p < 0.05) (Figure 5A, 5B, 5C). These data indicate treatment with DAPT restores osteogenic differentiation in MM-MSCs.

### Knockdown of Notch1 expression reverses osteogenic differentiation in MM-MSCs

**Figure 6:**
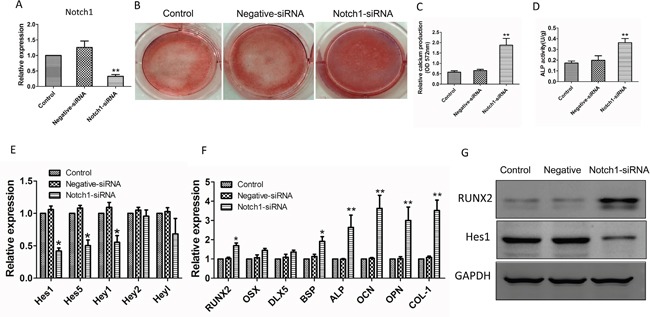
Knockdown of Notch1 expression reverses osteogenic differentiation in MM-MSCs **(A)** After 48h of Notch1 siRNA transfection, real-time PCR was performed to assess the mRNA expression of Notch1. **(B, C)** Notch1 knockdown significantly increased the mineralization of MM-MSCs after 21 days’ cultivation in osteogenic induction medium. **(D)** Notch1 knockdown significantly enhanced the ALP activity of MM-MSCs after cultured in osteogenic induction medium for 3 days. **(E)** Notch1 knockdown decreased the level of *hes-1*, *hes-5* and *hey-1* genes, compared to nontreated MM-MSCs or MM-MSCs transfected with negative control siRNA. **(F)** Notch1 knockdown increased the expression of *RUNX2*, *BSP*, *ALP*, *OCN*, *OPN* and *COL-1* genes involved in osteogenic differentiation in MM-MSCs after osteogenic induction for 7 days. **(G)** Notch1 knockdown resulted in a decreased protein level of hes-1 and an increased protein level of RUNX2 in MM-MSCs. Each experiment was repeated three times. All values are expressed as means ± SD. *p < 0.05, **p < 0.01.

MM-MSCs were transfected with Notch1 siRNA for 48 h to knockdown Notch1 expression. It was shown that Notch1 knockdown significantly increased the mineralization of MM-MSCs after 21 days’ cultivation in osteogenic induction medium and enhanced the ALP activity after 3 days (Figure 6B-6D). Notch1 knockdown decreased the level of *hes-1*, *hes-5* and *hey-1* genes, compared to nontreated MM-MSCs or MM-MSCs transfected with negative control siRNA (Figure 6E). In contrast, Notch1 knockdown increased the expression of *RUNX2*, *BSP*, *ALP*, *OCN*, *OPN* and *COL-1* genes involved in osteogenic differentiation in MM-MSCs after osteogenic induction for 7 days (Figure 6F). Western blot results showed *Notch1* knockdown resulted in a decreased protein level of hes-1 and an increased protein level of RUNX2 in MM-MSCs (Figure 6G). Collectively, knockdown of Notch1 expression reverses osteogenic differentiation in MM-MSCs.

### Activation of Notch signaling by NICD overexpression in BMMSCs induces impairment of osteogenesis

**Figure 7:**
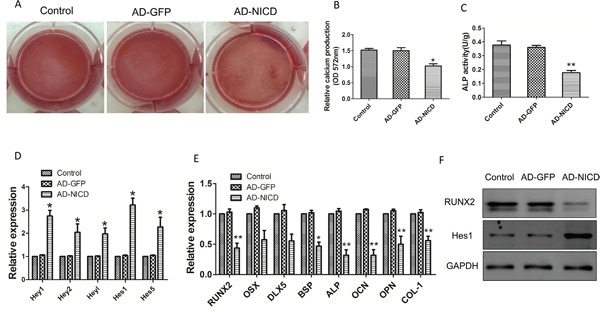
Activation of Notch signaling by NICD overexpression in HC-BMMSCs **(A, B)** Calcium production was reduced in the cells infected with NICD adenovirus after differentiation for 21 days, when compared to control cells. **(C)** ALP activity decreased in cells infected NICD adenovirus after osteogenic induction for 3 days, when compared to cells infected with GFP-control. **(D)** The mRNA expressions of Notch downstream genes (*hey-L, hey-1, hey-2 hes-1, and hes-5*) increased in BMMSCs infected with GFP-NICD. **(E)** The expression of *RUNX2*, *BSP*, *ALP*, *OPN* and *OCN* genes which were involved in osteogenic differentiation showed a decrease in BMMSCs infected with GFP-NICD, after osteogenic induction for 7 days, compared to control cells. **(F)** Overexpression of NICD induced the decreased protein expression of RUNX2. Each experiment was repeated three times. All values are expressed as means ± SD. *p < 0.05, **p < 0.01.

The adenovirus expressing Notch intracellular domain (NICD) (GFP-NICD) or GFP (GFP-control) were infected into normal BMMSCs. Calcium production was reduced in the cells infected with NICD adenovirus after differentiation for 21 days, when compared to control cells (Figure 7A, 7B). ALP activity decreased in cells infected NICD adenovirus after osteogenic induction for 3 days, when compared to cells infected with GFP-control (Figure 7C). The mRNA expressions of Notch downstream genes (*hey-L*, *hey-1*, *hey-2 hes-1*, and *hes-5*) increased in BMMSCs infected with GFP-NICD (Figure 7D). But the expression of *RUNX2*, *BSP*, *ALP*, *OPN* and *OCN* genes which were involved in osteogenic differentiation showed a decrease in BMMSCs infected with GFP-NICD, after osteogenic induction for 7 days, compared to control cells (*p<0.05) (Figure 7E). It was also shown that overexpression of NICD induced the decreased expression of RUNX2 by western blot (Figure 7F). These data indicate Notch signaling activation impairs osteogenesis of BMMSCs.

### Effects of lenalidomide (LEN) on the properties of BMMSCs

**Figure 8:**
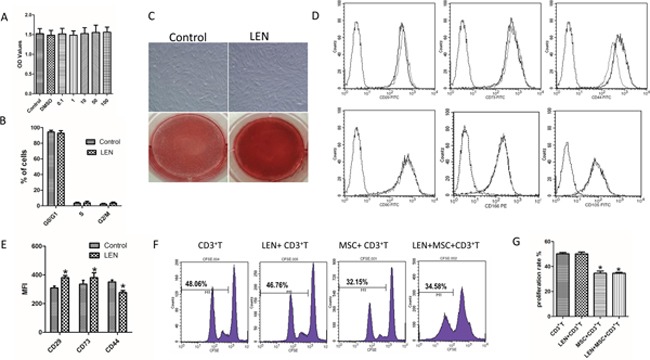
Effects of LEN on proliferation, phenotype, inhibition of T-cell proliferation, and differentiation capacity of BMMSCs **(A)** The proliferative capacity of BMMSCs was measured by CCK-8 assay after being cultured for 3 days in addition with or without LEN. It was not observed significant differences in the distinct HC-BMMSC preparations following LEN treatment (n=11). **(B)** No significant differences in cell cycle between LEN-treated group and control group were found. **(C)** LEN treatment did not affect the morphology of MSCs. Calcium production was promoted in the addition of LEN in BMMSCs after differentiation for 21 days. **(D, E)** LEN addition enhanced the mean fluorescence intensity (MFI) of CD29 and CD73 but suppressed the MFI of CD44 (dotted black line, isotype controls; solid gray line, LEN treated group; solid black line, untreated control;) (n= 12, *p < 0.05). **(F, G)** LEN addition had no significant effect on the proliferation of CD3^+^ T cells (*p* > 0.05). MSCs significantly inhibited the proliferation of CD3^+^ T cells both without (*p < 0.05) or with LEN addition (*p < 0.05).

BMMSCs were treated by LEN at 10 μM, then the phenotypes, proliferation and differentiation capacity, and the inhibition on T-cell proliferation were analyzed. The proliferation capacity of BMMSCs was measured by CCK-8 assay after being cultured for 3 days in addition with or without LEN (Figure 8A). It was not observed significant differences in the distinct HC-BMMSC preparations following LEN treatment. No significant differences in cell cycle between LEN-treated group and control group were found (Figure 8B). LEN treatment did not affect the morphology of MSCs (Figure 8C). However, calcium production was promoted in the addition of LEN in BMMSCs after differentiation for 21 days (Figure 8C). And LEN addition enhanced the mean fluorescence intensity (MFI) of CD29 and CD73 but suppressed the MFI of CD44 (Figure 8D, 8E). LEN addition had no significant effect on the proliferation of CD3^+^ T cells. MSCs significantly inhibited the proliferation of CD3^+^ T cells both without or with LEN addition (*p < 0.05) (Figure 8F, 8G).

### Impaired osteogenic differentiation of MM-MSCs is reversed by lenalidomide (LEN) via deactivating the Notch signaling pathway

**Figure 9:**
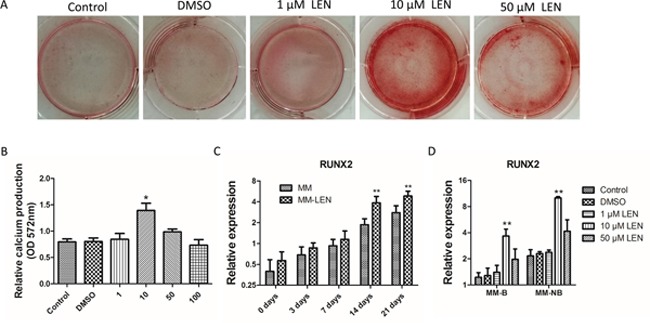
The influence of different concentrations of lenalidomide (LEN) on osteogenic differentiation of MM-MSCs **(A, B)** BMMSCs were exposed to LEN at different concentration (0, 1, 10, 50 and 100 μM) in the osteogenic differentiation medium. Relative calcium production by MM-MSCs was promoted with the addition of 10 μM LEN after differentiation for 21 days. **(C)** After 14 days of osteogenic differentiation, the mRNA expression of RUNX2 of 10 μM LEN treated MM-MSCs began to increase. **(D)** The mRNA expression of RUNX2 after 14 days of osteogenic differentiation with the treatment of different concentration (0, 1, 10, and 50μM). The results of MM-MSCs are shown separately as MM-B(n=7) and MM-NB(n=7). Results are expressed as mean±SD. *p<0.05, **p<0.01.

We further investigated whether LEN promotes osteoblast cell differentiation. Explant cultures of MM-MSCs derived from CD271-isolated primary MSCs were cultivated for 2 passages. BMMSCs were exposed to LEN at different concentration (0, 1, 10, 50, and 100 μM) in the osteogenic differentiation medium. First, LEN was added to the medium 3 days before the initiation of osteoblast cell culture. And osteogenic differentiation medium containing LEN were replenished every two days. As shown in Figure 9A, 9B, relative calcium production by MM-MSCs was promoted with the addition of 10 μM LEN after differentiation for 21 days. The mRNA level of *RUNX2* which was related with osteogenic differentiation was upregulated by 10 μM LEN treatment after osteogenic differentiation for 14 days (Figure 9C, 9D).

**Figure 10:**
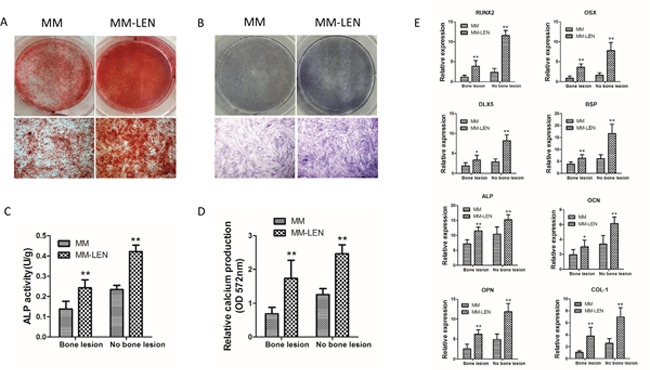
The influence of LEN on impaired osteogenic differentiation of MM-MSCs **(A)** Application of 10 μM LEN in the osteogenic differentiation medium increased mineralization evaluated by alizarin red S staining following 21 days of osteogenic induction. Representative original images and micrographs (original magnification, ×100) of MM-MSCs and MM-MSCs treated with LEN are shown. **(B)** BMMSCs from MM patients (MM) and MM-patients with LEN treatment (MM-LEN) were evaluated for their osteogenic differentiation potential by ALP staining after 3 days of osteogenic culture. ALP staining was performed with BCIP/NBT. Representative original images and micrographs (original magnification, ×100) of MM-MSCs and MM-MSCs treated with LEN are shown. **(C)** After 3 days of differentiation in osteogenic medium, ALP activity showed an increase in BMMSCs (derived from MM-B and MM-NB) treated with LEN (n=5, **p< 0.01). **(D)** Relative calcium production by BMMSCs from MM-B and MM-NB was enhanced with the addition of LEN after differentiation for 21 days (n=6, **p<0.01). **(E)** Expression of genes, *OSX*, *DLX5*, *RUNX2*, *BSP*, *ALP*, *OCN*, *OPN*, and *COL-1* were upregulated by LEN after osteogenic differentiation for 14 days. The results of MM-MSCs are shown separately as MM-B(n=7) and MM-NB(n=7). All values are expressed as means ± SD. *p < 0.05, **p < 0.01.

**Figure 11:**
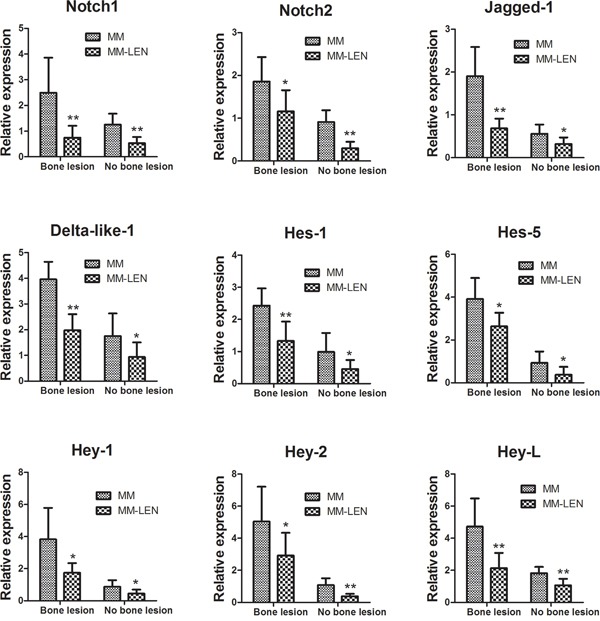
The effect of LEN on the expression of genes associated with Notch signaling in MM-MSCs Relative mRNA expression of *Notch1*, *Notch2*, *Hes-1*, *Hes-5*, *Jagged-1*, and *Deltalike-1* were significantly decreased in MM-MSCs treated with LEN as compared with MM-MSCs untreated. MM-MSCs are shown separately as MM-B(n=7) and MM-NB(n=9). All values are expressed as means ± SD. *p < 0.05, **p < 0.01.

Therefore, the lenalidomide at 10 μM was applied for the following assays according to a dose-response curve with concentrations at 0, 1, 10, 50, and 100 μM. As shown in Figure 10C, after 3 days of differentiation in osteogenic medium, ALP activity showed an increase in BMMSCs (derived from MM-B and MM-NB) treated with LEN (**p< 0.01). Relative calcium production by BMMSCs from MM-B and MM-NB was enhanced with the addition of LEN after differentiation for 21 days (Figure 10D). And it also was observed that the expression of genes, *OSX*, *DLX5*, *RUNX2*, *BSP*, *ALP*, *OCN*, *OPN*, and *COL-1* were upregulated by LEN after osteogenic differentiation for 14 days (Figure 10E), but mRNA expression of *Notch1*, *Notch2*, *Hes-1*, *Hes-5*, *Jagged-1*, and *Deltalike-1* were significantly decreased in MM-MSCs treated with LEN as compared with MM-MSCs untreated (*p<0.05) (Figure 11). Taken together, the data indicate that impaired osteogenic differentiation of MM-MSCs is reversed by lenalidomide (LEN) treatment via the attenuated activation of Notch signaling pathway.

## DISCUSSION

The mechanisms of bone lesions in MM remain unexplored. It is ambiguous that whether the defects in bone formation and repairment result from the dysfunction of stromal progenitors, the decrease in mature differentiated osteoblasts, the failure in response to osteogenic signals or the combination [24, 26, 27]. We explored the possible mechanisms by systematically analyzing BMMSCs from MM patients or healthy controls and compared their osteogenic ability. CD271^-^ALP^+^ OB show obvious abatement in primary BMMSCs from MM patients, exhibiting the significantly impaired osteogenesis. Previous studies report unconsistent results about the production of BMMSC in MM patients compared to healthy controls. In this research, we find the frequency of BMMSCs and osteoblast progenitor in freshly isolated BM from MM patients is different from healthy controls. In addition, the number of CD271^-^ALP^+^ OB from MM patients are significantly lower in MM patients with bone lesion, old age, del 17p, and higher CD138^+^ PC burden. The generated MM plasma cells probably act on the resident BMMSCs to inhibit osteogenesis, leading to the disruption of the system.

It is that observed MM-MSCs are not capable of conduct the osteoblast programme. The expression of transcription factors *OSX*, *RUNX2*, and *DLX5* are strikingly downregulated in MM before osteogenic differentiation. The level of the genes expressed in early differentiation (*ALP*, *BSP*, and *COL-1*) or late differentiation (*OPN* and *OCN*) are much more lower in MSC from MM than healthy controls after osteogenic induction during differentiation toward mature osteoblasts. But the OB differentiation markers (*DLX5* and *COL-1*), indicating the early osteogenic differentiation process, do not show significance change between healthy controls and patients after differentiation for 21 days. Arnulf et al report that BMMSCs from MM patients have normal phenotype, hematopoietic support and differentiation ability [7]. Kassen et al find that potential and frequency of osteoblast progenitor and MSC is similar in acutely isolated bone marrow from MM patients with age-matched controls [26]. Conversely, Xu et al observe a decrease in ALP generation by MSCs from MM patients than control 72 h after osteogenic differentiation [28], while it is also reported that MM-MSCs are not observed abnormality [5]. Such inconsistent results probably are because of variations in experimental conditions. Collectively the available data indicate MM-MSCs have a defect in osteogenesis.

Our results confirm that BMMSCs in patients with MM have diminished osteogenic differentiation, presented as attenuated level of genes involved in osteogenic differentiation and relative calcium production. A growing data has demonstrated the elevated expression of the relevant genes of Notch signaling pathway in many hematopoietic malignant cells [8–10]. Several researches report that Notch signaling maintain BMMSCs in the undifferentiated state by suppressing osteoblast differentiation [11, 13–15]. The present study show the level of Notch ligands *Deltalike-1*, *Jagged-1* are much more higher in MM-MSCs compared to controls, which are consistent with the data in paper from XU et al [28]. It is also shown the expression of *Notch1*, *Notch2*, *Hes-1*, *Hes-5* are altered in MM-MSCs. In bone marrow, Notch signaling suppresses the osteogenic differentiation via inhibiting *RUNX2* transcriptional activity to play important roles in maintaining the pool of MSCs. Our study shows addition of DAPT or Notch1 siRNA reduces the expression of hes1 and elevates the level of *RUNX2* in MM-MSCs. Moreover, inhibition of Notch signaling induces the enhancement of osteogenic genes, enhanced ALP activity and mineralization deposition, when compared with control. We also find that activation of Notch signaling by overexpression of NICD obviously impairs osteogenic differentiation potential characterized by a reduction in the expression of osteoblastic markers, ALP activity and calcium deposits in HC-MSCs, indicating that activated Notch signaling contributes to the abnormal osteogenic differentiation in MM. Some drugs targeting Notch signaling are already tested in clinical trials for treatment in cancer. Inhibition of Notch activity in cancer stem cells promotes their differentiation to inhibit the formation of tumor mass. Inhibitor of Notch might be an attractive drug to facilitate osteogenesis and modulate BM microenvironment.

The effective therapy of MM needs to be further investigated. Novel drugs to target MM and microenvironment have shown promising clinical effects. It is reported that bortezomib, the first approved member of proteasome inhibitors for the clinical application, affects human osteoblast differentiation process both *in vitro* and *in vivo* [17]. Bortezomib treatment increases RUNX2/Cbfa1 activity to stimulate the expression of late osteogenic markers, OC and collagen I, rather than early ones as ALP and therefore consequently induces osteoblast progenitors into osteoblast [17]. One study shows lenalidomide affects the activation of osteoclasts, osteoclast genesis, related growth factors, and markers of bone turnover in MM [19]. So far data about the effects of lenalidomide on osteogenesis are very limited and display contradictionary effect. Shoso et al show that 10 μM LEN addition inhibits osteoclastogenesis without suppressing osteogenic differentiation [29]. But in another study, it is observed LEN treatment leads to higher ALP activity in some MSCs after differentiated for 21 days [20]. Our present study show that relative calcium production by MM-MSCs was promoted in the addition of LEN osteogenic differentiation for 21 days. And ALP activity was significantly enhanced in osteogenic induction medium following LEN application after differentiation for 7 days. Furthermore, it is found that the expression of the osteogenic gene such as *OSX*, *DLX5*, *RUNX2*, *BSP*, *ALP*, *OCN*, *OPN*, and *COL-1* is upregulated by LEN after osteogenic differentiation for 7 days and LEN treatment increases MFI of CD29 and CD73, with the decrease in MFI of CD44. The variable results may result from the universally high heterogeneity of primary MSCs, differences in isolated MSCs, culture methods and the time point or concentration of LEN used. Besides, our study show that treatment of MSCs with LEN has no effects on the growth rate, proliferation, or ability to restrain proliferation of T-cell.

## MATERIALS AND METHODS

### Patients

This study was approved by the Ethics Committee of Shanghai Jiao Tong University Affiliated Sixth People's Hospital (Shanghai, China) and each subject provided written informed consent. Forty-eight consecutive patients with newly diagnosed MM (n=48) were enrolled between November 2014 and December 2016 (Table 1). All patients were diagnosed according to the International Myeloma Working Group criteria [30], and were staged according to the criteria of ISS [31]. Twenty-four healthy controls with a median age of 56 years (range, 24-68 years) were investigated in our research.

**Table 1:** Clinical characteristics of patients with multiple myeloma

Characteristic	Bone diseaseN= 18	No bone diseaseN=30
Sex Male, n (%)	10(56%)	16(53%)
Female, n (%)	8(44%)	14(47%)
Median age, years (Range)	66(48-80)	63 (36-88)
Immunoglobuline0 subtype, n (%)		
IgG	8(44%)	16(53%)
IgA	5(28%)	7(23%)
Light-chain		
κ	2(11%)	3(10%)
λ	1(6%)	2(7%)
Non-secretory	2(11%)	2(7%)
International staging system, n (%)		
I	0(0%)	4(13%)
II	3(17%)	21(70%)
III	15(83%)	5(17%)
Plasma cell infiltration, median (Range)	53%(32%-87%)	21.25%(9.8%-51%)
Medianβ2-microglobulin, mg/L (Range)	6.51(3.6-15.07)	3.9(1.8-6.3)
Median hemoglobin, g/dL (Range)	8.1(4.9-12.6)	10.6(6.6-16.3)
Median creatinine, μmol /L (Range)	103(65-218)	69(54-112)
Median calcium, mmol/L (Range)	2.86(2.45-3.76)	2.3(1.93-2.45)
Median albumin, g/dL (Range)	4(2.3-4.6)	3.6(2.6-6.8)
Median LDH, U/L (Range)	332(206-603)	149(108-227)
Cytogenetic aberrancies, n (%)		
P53(17P13)	12(67%)	7(23%)
IgH (14q32)	10(56%)	6(20%)
D13S319(13q14.3)	13(72%)	8(27%)
1q21(1q21)	10(56%)	12(40%)
RB1(13q14)	13(72%)	9(30%)

### Primary mesenchymal stem cells

Density gradient centrifugation was performed to obtain bone marrow mononuclear cells (BMMNCs). CD271^+^ cells were selected using MACS separation kit (Miltenyi Biotec, Bergisch Gladbach, Germany). CD271^+^-selected cells were cultured in 6-well plates at low density for 10 days and analyzed by flow cytometry for the expression of CD271 and alkaline phosphatase (ALP, TRA-2-49). The monoclonal antibodies are CD271-FITC (Miltenyi Biotec, Bergisch Gladbach, Germany) and TRA-2-49-PE (Biolegend, San Diego, CA, USA).

### Isolation, culture, osteogenic differentiation and immunophenotype of BMMSCs

BMMSCs were isolated, cultured and induced into osteoblast. The procedure was detailed in our previous studies [32, 33]. The immunophenotype of BMMSCs were evaluated at P3 (the third passage). After 15 min incubation with the antibodies as follow: CD105-FITC, CD73-FITC, CD29-FITC, CD90-FITC, CD166-PE, CD34-PE, CD45-FITC, CD80-FITC, CD86-FITC (BD Biosciences Pharmingen, San Diego, California, USA), CD44-FITC (Miltenyi Biotec, Bergisch Gladbach, Germany), BMMSCs were analyzed by FACS Calibur system (BD, Mountain View, CA, USA) using Cell Quest software.

### Lenalidomide treatment

Lenalidomide (Selleck, Shanghai, China) was stored at -20°C after dissolution in 10% dimethyl sulfoxide(DMSO). Based on the results of the dose-dependent experiments and the data of del (5q) MDS and MM [20, 34–36], 10 μM lenalidomide (LEN) was chosen for the effect on immunophenotype, proliferation, differentiation capacity and inhibition of CD3^+^T cells. Primary MSCs (CD271^+^ selected and expanded) were exposed to LEN of different concentration (0, 1, 10, 50, and 100 μM) in the osteogenic differentiation medium. And the medium was replenished every two days and LEN was added.

### Detection of osteogenesis

After culture in osteogenic induction medium for indicated time, cells were analyzed for alkaline phosphatase (ALP) activity, mineralization and ALP staining. The methods for detecting ALP activity and mineralization were described in our article previously [33]. ALP staining was performed with BCIP/NBT (Sigma Aldrich, St Louis, MO, USA). BCIP/NBT was added to the cells and incubated for 1 hour. Microscopically, the dark purple cells were considered as ALP positive.

### Lymphocyte proliferation assay

CD3^+^ T cells were isolated by using MACS separation kit (Miltenyi, Auburn, CA, USA). CD3^+^T cells stained with carboxyfluorescein diacetate succinimidyl ester (CFSE; Invitrogen, Carlsbad, CA, USA) were co-cultured with BMMSCs. The method was detailed in our previous article [37].

### Real-time PCR

Total RNA was isolated using the RNeasy Mini Kit (Qiagen, Germany) according to the manufacturer's instructions. For mRNA detection, reverse transcription of RNAwas performed using the ReverTra Ace qPCR RT Kit (TOYOBO, Osaka, Japan), and real-time PCR (RT-PCR) was carried out using SYBR® Premix Ex Taq™ II (Tli RNaseH Plus) (Takara, Kusatsu, Shiga, Japan). The primers are listed in Table 2. The process was detailed in our previous study [33].

**Table 2:** The sequence of primers used for real time PCR

Gene	Forward primer (5' - 3')	Reverse primer (5' - 3')
GAPDH	GCACCGTCAAGGCTGAGAAC	GTGGTGAAGACGCCAGTGGA
RUNX2	AGTGGACGAGGCAAGAGTTTC	CCTTCTGGGTTCCCGAGGT
Osterix	TCCTCCTGCGACTGCCCTAA	TGCGAAGCCTTGCCATACA
ALP	CCATTCCCACGTCTTCACATT	AAGGGCTTCTTGTCTGTGTCACT
BSP	GACAGTTCAGAAGAGGAGGAG	AGCCCAGTGTTGTAGCAGA
OCN	AGGGCAGCGAGGTAGTGAA	TCCTGAAAGCCGATGTGGT
OPN	TTTACAACAAATACCCAGATGC	ATGGCTTTCGTTGGACTTACT
COL-1	CACCAATCACCTGCGTACAGAA	CAGATCACGTCATCGCACAAC
DLX5	TTCCAAGCTCCGTTCCAGAC	GAATCGGTAGCTGAAGACTCG
Notch1	CTTGTGTCAACGGCGGC	TTGGGACCGCTGAAGCC
Notch2	GGCCACCTGAAGGGAAGCACATA	CACAGAGGCTGGGAAAGGATGATA
Hes1	AGGCTGGAGAGGCGGCTAAG	TGGAAGGTGACACTGCGTTGG
Hes5	ACCAGCCCAACTCCAAGCT	GGCTTTGCTGTGCTTCAGGTA
Hey1	GGATCACCTGAAAATGCTGCATAC	CCGAAATCCCAAACTCCGATAG
Hey2	GAACAATTACTCGGGGCAAA	TCAAAAGCAGTTGGCACAAG
HeyL	AGCCAGGAAGAAACGCAGAGG	GCTGTTGAGGTGGGAGAGAAGG
Deltalike1	TCCTGATGACCTCGCAACAGA	ACACACGAAGCGGTAGGAGT
Jagged1	TCGGGTCAGTTCGAGTTGGA	AGGCACACTTTGAAGTATGTGTC

### DAPT treatment

During the osteogenic differentiation of MM-MSCs, DAPT (Sigma, Saint Louis, MO, USA), which inhibits γ-secretase, was added to the culture medium. DAPT was dissolved in DMSO (Sigma, Saint Louis, MO, USA). The final concentration of DAPT in the medium was 10 μM. The cells treated with DMSO served as controls and all cells were harvested after 48 hours.

### Notch1 knock-down by RNA interference

To knock down the expression of Notch1, MM-MSCs were transfected with siRNA for Notch1. FlexiTube Gene Solution GS4851 for Notch1 (Qiagen, Germany), which contains 4 non-overlapping siRNAs for Notch1 to achieve high knock-down efficiency was used. MM-MSCs were transfected with Notch1 siRNA with the final concentration at 20nM by using Lipofectamine RNAiMAX (Invitrogen, USA) on the basis of the instructions. We set All Stars Negative Control siRNA (Qiagen, Germany) as negative control. After 48 hours of Notch1 siRNA transfection, real-time PCR was applicated to evaluate the efficiency of Notch1 knock-down.

### Adenovirus mediated cell transfection

The process of the construction of adenoviral vectors for expression of NICD was detailed previously [32, 38]. The HC-MSCs were planted in 6-well plates and cultivate for 24 hours. The cells were transfected with 20 μl adenoviral vectors and cultured for 12 hours and then the medium was changed. The cells were cultured for another 36 hours and harvested.

### Western blotting analysis

The cellular lysate was fractionated by 12% SDS-PAGE and electroblotted onto PVDF membranes. Membranes were incubated with primary antibodies including anti-Hes1 (rabbit, Abcam, USA), anti-RUNX2 (rabbit, Abcam, USA) and anti-GAPDH (mouse, Abcam, USA). Secondary goat anti-mouse or anti-rabbit antibodies labeled with horseradish peroxidase (Amersham Biosciences) were used. The process was detailed in our previous study [32, 38].

### Statistical analysis

Statistical analyses were conducted using SPSS software version 17.0. Two-sample t-tests were used to compare two independent samples. Multiple pairwise comparisons were made using a one-way analysis of variance (ANOVA). A value of *p*<0.05 was considered statistically significant.

## CONCLUSIONS

The present study demonstrates that the osteogenic differentiation is impaired in BMMSCs from patients with MM. Treatment with lenalidomide restores the osteogenic differentiation of bone marrow mesenchymal stem cells from MM patients via deactivating Notch signaling pathway.
